# Idiopathic hypereosinophilic syndrome in remission with benralizumab treatment after relapse with mepolizumab

**DOI:** 10.1002/rcr2.665

**Published:** 2020-09-23

**Authors:** Koki Fujii, Hidenori Takahashi, Nami Hayakawa, Yoshinobu Iwasaki

**Affiliations:** ^1^ Division of Respiratory, Department of Medicine Showa General Hospital Tokyo Japan; ^2^ Department of Plastic Surgery Tokyo Women's Medical University Hospital Tokyo Japan

**Keywords:** Benralizumab, corticosteroid‐sparing effect, idiopathic hypereosinophilic syndrome, IL‐5, mepolizumab

## Abstract

We report a patient with idiopathic hypereosinophilic syndrome (I‐HES) who achieved remission with benralizumab after relapsing on mepolizumab. An 83‐year‐old man was admitted to Showa General Hospital after presenting with hypoxaemia and multiple erythematous lesions. He showed a marked increase in blood eosinophil count. Skin biopsy revealed an invasion of eosinophils in the dermis. He was diagnosed with I‐HES. He was commenced on prednisolone 40 mg/day with a plan to wean this over time after pulse steroid therapy for three days. Mepolizumab was added when the prednisolone dose was 25 mg/day. Unfortunately, at a prednisolone dose of 5 mg/day, there was evidence of disease progression and the patient was switched to benralizumab. Prednisolone was tapered again and, finally, the patient was in remission. Benralizumab targets interleukin (IL)‐5R and induces antibody‐dependent cell‐mediated cytotoxicity, thereby reducing the eosinophil counts in the tissue. This can be attributed to the therapeutic efficacy against I‐HES. We believe this report may help develop novel therapeutic strategies for I‐HES.

## Graphical abstract

We report a patient with idiopathic hypereosinophilic syndrome (I‐HES) who achieved remission with benralizumab after relapsing on mepolizumab. We believe this report may help develop novel therapeutic strategies for I‐HES.

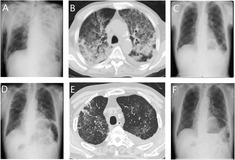

## Introduction

Hypereosinophilic syndrome (HES) comprises a group of diseases that are characterized by prolonged peripheral eosinophilia and organ damage in the absence of secondary causes for eosinophilia. Recent reports have shown myeloproliferative, lymphoproliferative, overlap, idiopathic, and associated variants of HES. The recent past has seen the evolution of therapeutic strategies for HES.

Idiopathic HES (I‐HES) is primarily treated using corticosteroids. However, corticosteroids are associated with adverse events and symptomatic relapse and need to be discontinued. Prednisolone has been discontinued in 42% of patients due to lack of efficacy, intolerance, or other reasons in a study [1].

Monoclonal antibodies targeting interleukin (IL)‐5 or IL‐5R have corticosteroid‐sparing effect and they are analysed as part of ongoing clinical trials. Antibodies have specific receptor targets. Benralizumab and mepolizumab have separate and complementary mechanisms of action useful for the treatment of I‐HES. There are a few reports of benralizumab being used to treat I‐HES after relapse with mepolizumab. A study has described the use of benralizumab in a phase II trial. Only three HES patients have been treated (with one achieving remission) with benralizumab after mepolizumab in the phase II trial [2].

## Case Report

**Figure 1:**
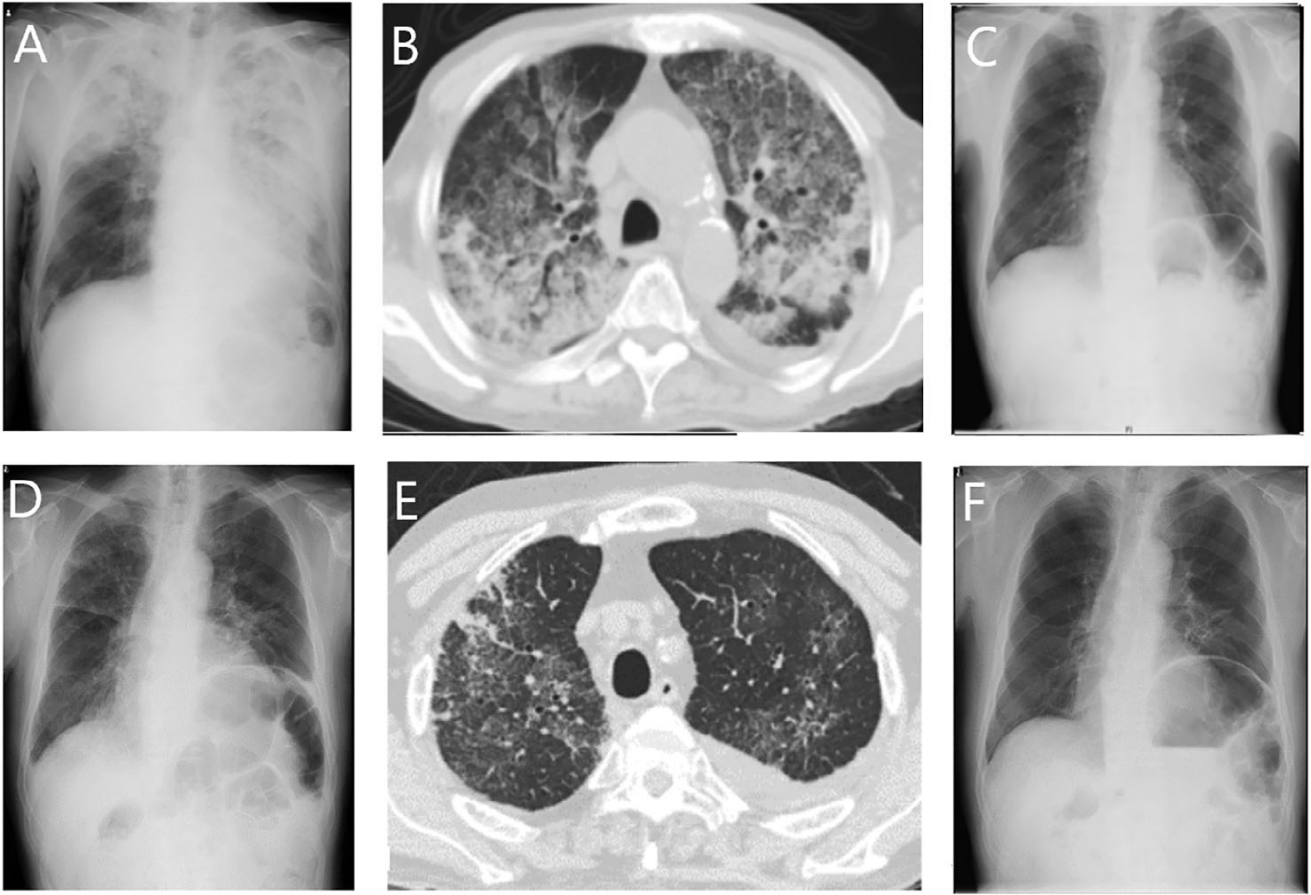
(A) Chest radiograph after the patient's hospitalization revealed a consolidation in the left lung and right upper lung. (B) Computed tomography on hospitalization showed a mixed pattern of air‐space consolidation, air bronchogram, and ground‐glass opacities. (C) Chest radiograph at discharge showed improvement in consolidation. (D) Chest radiograph at readmission showed ground‐glass opacities and pleural effusion in both lungs. (E) Computed tomography at readmission also showed ground‐glass opacities and pleural effusion in both lungs. (F) Chest radiograph after benralizumab therapy showed improvement of ground‐glass opacities and pleural effusion.

An 83‐year‐old male was hospitalized five months after being recovered from pneumonia. His blood eosinophil level was ~10 × 10^3^/μL. He was admitted to Showa General Hospital after presenting with hypoxaemia and multiple erythematous lesions. Physical examination during his initial visit revealed that he was 165.0 cm and 60.7 kg with a body temperature of 38.6°C and had a blood pressure of 138/84 mmHg, radial pulse rate of 106/min, respiratory rate of 28/min, and peripheral capillary oxygen saturation (SpO_2_) of 75% (room air)–92% (6 L/min oxygen). There were multiple erythematous lesions throughout his whole body. Fine crackles were heard in both his lungs upon auscultation. Laboratory work‐up revealed leucocytosis with 21.8 × 10^3^/μL, 48.0% eosinophils, and C‐reactive protein levels of 12.88 mg/dL. Chest radiographs showed consolidation mainly in the left lung and right upper lung (Fig. 1A). Computed tomography showed a mixed pattern of air‐space consolidation, air bronchogram, and ground‐glass opacities (Fig. 1B). Skin biopsy revealed significant infiltration of eosinophils around the blood vessels in the dermis. His peripheral blood was negative for FIP1L1‐PDGFRA with normal levels of serum immunoglobulin (Ig) E and Ig antinuclear antibodies. He was negative for MPO‐ANCA and PR3‐ANCA. He did not have sinus disease or nasal polyps. He was negative for HIV five years ago. Other possible causes of hypereosinophilia, such as parasite infection and drugs, were not detected. He was diagnosed with I‐HES based on Chusid's criteria.

**Figure 2:**
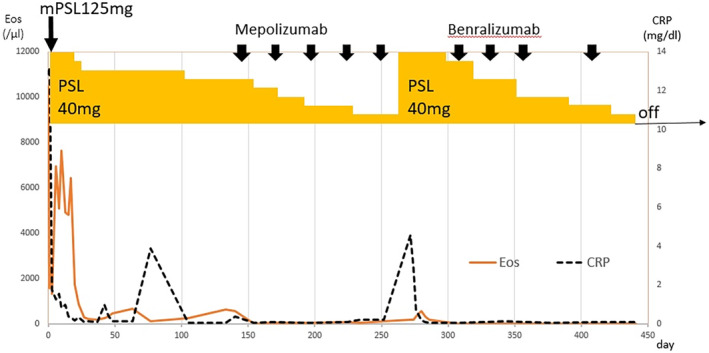
Clinical course of the patient with idiopathic hypereosinophilic syndrome on administering the monoclonal antibodies. CRP, C‐reactive protein; Eos, Eosinophil; mPSL, methylprednisolone; PSL, prednisolone.

Figure 2 shows the clinical course of the patient. He was commenced on prednisolone 40 mg/day with a plan to wean this over time after pulse steroid therapy for three days. His blood eosinophil count decreased, thereby improving the state of disease. He was prescribed a daily dose of 30 mg of prednisolone and discharged (Fig. 1C). Mepolizumab was added when the prednisolone dose was 25 mg/day. Unfortunately, at a prednisolone dose of 5 mg/day, there was evidence of disease progression. He was readmitted for hypoxaemia, SpO_2_ of 90% (room air), and multiple erythematous lesions. Chest radiographs and computed tomography showed ground‐glass opacities and pleural effusion in both lungs (Fig. 1D, E). We diagnosed recurrence and the dose of prednisolone was increased to 40 mg/day, thereby improving the disease. The patient was switched to benralizumab. His blood eosinophil count was almost 0/μL and the disease has been in remission for about two years (Fig. 1F).

## Discussion

Mepolizumab is a humanized monoclonal antibody that targets IL‐5 and selectively inhibits eosinophilic inflammation while reducing eosinophil content in the sputum and blood. According to a multicentre placebo‐controlled trial of mepolizumab in patients with PDGFRA‐negative HES, mepolizumab was well tolerated and had a significant glucocorticoid‐sparing effect. However, this holds true only in 50% of the airway tissues and bone marrows and has no significant effect on bronchial mucosal staining of the eosinophil major basic protein [3].

Benralizumab is an anti‐human IL5‐Rα antibody that inhibits ligand‐independent IL‐5 receptor signalling. It produces antibody‐directed cell‐mediated cytotoxicity against eosinophils and basophils and depletes IL‐5Rα‐expressing cells. Blocking IL‐5R signalling independent of a ligand and depleting IL‐5Rα‐expressing cells enhance clinical efficacy. Benralizumab treatment depletes eosinophils from the peripheral blood more than that using mepolizumab. Benralizumab depletes eosinophils in the airway mucosa, sputum, and bone marrow. Benralizumab produces a median decrease in the content of peripheral blood eosinophils from a baseline of 95.8% in the airway mucosa, 89.9% in sputum, and 100% in the bone marrow [4].

The life cycle of eosinophils comprises three phases: bone marrow, blood, and tissue phase. They are primarily tissue‐dwelling cells. Once eosinophils have entered the blood, they have a short half‐life ranging from 8 to 18 h. After circulating in the blood, eosinophils migrate into the tissues, where the life span ranges from two to five days. In humans, the eosinophil tissue:blood ratio is ~100:1.14; thus, eosinophils merely “pass through” circulation en route to the tissues [5].

Taken together, benralizumab, not mepolizumab, was effective in depleting tissues of their eosinophils. Benralizumab was more effective in treating I‐HES by targeting IL‐5R rather than IL‐5. This will help develop effective corticosteroid‐sparing treatment methods based on benralizumab alone or in combination with mepolizumab. Future studies should focus on identifying and devising therapeutic interventions that are more efficacious for I‐HES.

### Disclosure Statement

Appropriate written informed consent was obtained for publication of this case report and accompanying images.

## References

[1] Ogbogu PU , Bochner BS , Butterfield JH , et al. 2009 Hypereosinophilic syndrome: a multicenter, retrospective analysis of clinical characteristics and response to therapy. J. Allergy Clin. Immunol. 124(6):1319–1325. 19910029 10.1016/j.jaci.2009.09.022 PMC2829669

[2] Kuang FL , Legrand F , Makiya M , et al. 2019 Benralizumab for *PDGFRA*‐negative hypereosinophilic syndrome. N. Engl. J. Med. 380(14):1336–1346. 30943337 10.1056/NEJMoa1812185 PMC6557265

[3] Flood‐Page PT , Menzies‐Gow AN , Kay AB , et al. 2003 Eosinophil's role remains uncertain as anti‐interleukin‐5 only partially depletes numbers in asthmatic airway. Am. J. Respir. Crit. Care Med. 167(2):199–204. 12406833 10.1164/rccm.200208-789OC

[4] Laviolette M , Gossage DL , Gauvreau G , et al. 2013 Effects of benralizumab on airway eosinophils in asthmatic patients with sputum eosinophilia. J. Allergy Clin. Immunol. 132(5):1086–1096. 23866823 10.1016/j.jaci.2013.05.020 PMC4172321

[5] Park YM , and Bochner BS . 2010 Eosinophil survival and apoptosis in health and disease. Allergy Asthma Immunol. Res. 2(2):87–101. 20358022 10.4168/aair.2010.2.2.87 PMC2846745

